# Evaluation of blood gene expression levels in facioscapulohumeral muscular dystrophy patients

**DOI:** 10.1038/s41598-020-74687-5

**Published:** 2020-10-16

**Authors:** M. Signorelli, A. G. Mason, K. Mul, T. Evangelista, H. Mei, N. Voermans, S. J. Tapscott, R. Tsonaka, B. G. M. van Engelen, S. M. van der Maarel, P. Spitali

**Affiliations:** 1 Department of Biomedical Data Sciences, Leiden University Medical Center, 2333 ZC Leiden, The Netherlands; 2 Department of Human Genetics, Leiden University Medical Center, 2333 ZC Leiden, The Netherlands; 3 Department of Neurology, Donders Institute for Brain, Cognition and Behaviour, Radboud University Medical Center, Nijmegen, The Netherlands; 4 John Walton Muscular Dystrophy Research Centre, Institute of Genetic Medicine, Newcastle University, Centre for Life, Newcastle, NE1 3BZ UK; 5 Sorbonne Université, AP-HP, INSERM, Centre de référence Des Maladies Neuromusculaires Nord/Est/Ile de France, Groupe Hospitalier Universitaire La Pitié-Salpêtrière, Paris, France; 6 Human Biology Division, Fred Hutchinson Cancer Research Center, Seattle, WA 98109 USA; 7 Department of Neurology, University of Washington, Seattle, WA 98105 USA

**Keywords:** Genetics, Biomarkers, Diseases, Medical research

## Abstract

Facioscapulohumeral muscular dystrophy (FSHD) is caused by the expression of *DUX4* in skeletal muscles. A number of therapeutic approaches are being developed to antagonize the events preceding and following *DUX4* expression that leads to muscular dystrophy. Currently, the possibility to evaluate treatment response in clinical trials is hampered by the lack of objective molecular biomarkers connecting the disease cause to clinical performance. In this study we employed RNA-seq to examine gene expression in PAXgene tubes obtained from two independent cohorts of FSHD patients. Analysis of gene expression profiles did not lead to the identification of genes or pathways differentially expressed in FSHD patients, or associated with disease severity. In particular, we did not find evidence that the *DUX4* and *PAX7* signatures were differentially expressed. On the other hand, we were able to improve patient classification by including single genes or groups of genes in classification models. The best classifier was *ROPN1L*, a gene known to be expressed in testis, coincidentally the typical location of *DUX4* expression. These improvements in patient classification hold the potential to enrich the FSHD clinical trial toolbox.

## Introduction

Facioscapulohumeral muscular dystrophy (FSHD) is caused by expression of the *DUX4* gene in skeletal muscle because of D4Z4 macrosatellite repeat chromatin de-repression. Transcription prone D4Z4 chromatin conformation is the result of repeat contraction to a size of 1–10 units (FSHD type 1; FSHD1) or due to variants in the genes encoding the D4Z4 chromatin modifiers SMCHD1, DNMT3B or LRIF1 (FSHD type 2; FSHD2)^1–5^. Stable expression of *DUX4* in somatic cells depends on the presence of a somatic polyadenylation site in exon 3 of the *DUX4* locus situated immediately distal to the last repeat of the D4Z4 array. The effect of *DUX4* expression on FSHD pathogenesis has been reported in cultured patient derived cells and muscle biopsies^6^. The detrimental effect of *DUX4* on myogenesis and terminal differentiation is further corroborated by different types of cancer, specifically fusion sarcomas, where nuclear *DUX4* expression is considered a promising histochemical marker^7–9^.

The discovery of the disease cause sparked the investigation into therapeutic approaches to reduce *DUX4* expression via antisense oligonucleotides^10^, *DUX4* targeting miRNAs^11,12^, CRISPR mediated DUX4 repression^13^, repair of the *SMCHD1* pathogenic variants^14^ and overexpression of SMCHD1^15^. Other approaches include, but are not limited to, *DUX4* decoy molecules^16^ and molecules targeting the pathophysiologic effect resulting from *DUX4* expression^17^.

Despite strong evidence collected in vitro, investigation of *DUX4* mRNA and protein have proven challenging due to rapid mRNA and protein degradation^18^ and their heterogeneous expression, thereby posing a challenge to assess the drug pharmacodynamic (PD) effects of *DUX4* targeting approaches in pre-clinical studies as well as in up-coming clinical trials. To compensate for this analytical challenge, it has been proposed to use the *DUX4* and *PAX7* signatures as surrogate readouts for *DUX4* expression^19–22^. Indeed, the *DUX4* activated *DUX4/PAX7* gene signature has been shown to remain active for a longer time in cultured cells^23^, suggesting that it could be exploited as a PD biomarker in interventional trials aiming to lower or to prevent *DUX4* expression. While these observations are mainly based on experimental evidence obtained in cultured cells and muscle biopsies, their use in clinical trials could be affected by the availability of muscle biopsies, and by the analytical performance of the methods used to detect these signatures. Given the low expression of *DUX4* in muscle, the ability to show target engagement in clinical trials remains limited. Aside from the identification of PD biomarkers for *DUX4* lowering approaches, drug developers will need to prove that there is a clinically meaningful benefit, irrespective of the drug type and of its mechanism of action. The slow progressive nature of FSHD^24^, combined with often asymmetric and variable clinical presentation, constitutes a further challenge to evaluate drug related efficacy. So far, small-scale cross-sectional comparisons of serum samples allowed the identification of proteins discriminating between FSHD and healthy controls; however, the signature was not connected to *DUX4* expression and the context of use was not clearly specified^25,26^. Identification of serum biomarkers with clearly defined context of use would be of great support for drug developers. While prognostic and predictive capacity could contribute to enrich clinical trial design, pharmacodynamic biomarkers would be able to support claims of target engagement in interventional studies.

In this study we collected blood of FSHD patients in PAXgene tubes at two different sites. Analysis of the samples by RNA sequencing (RNA-seq) was performed to identify RNAs that could be used to separate FSHD patients from healthy controls, and to propose biomarkers for *DUX4* lowering approaches. Association of blood biomarkers with patients’ performance was also explored to identify potential biomarkers that could be used to support claims of efficacy in clinical trials. Overall, the results presented in this article show that the blood signature present in FSHD is weaker than that observed for other muscular dystrophies such as Duchenne Muscular Dystrophy (DMD)^26, 27^. No clear association with the *DUX4*/*PAX7* signatures and scores was identified, suggesting that investigation of RNA species in blood does not enable direct evaluation of PD effects for *DUX4* lowering drugs in small patient populations with the setup used in this study.

## Materials and methods

### Recruitment and characteristics of the subjects involved in the study

Patients involved in this study were recruited from two hospitals, the Radboud University Medical Center (hereafter referred to as RUMC) in Nijmegen, The Netherlands, and the John Walton Muscular Dystrophy Centre of the University of Newcastle (hereafter UNEW) in Newcastle, United Kingdom. A total of 83 blood samples were collected in PAXgene tubes from 54 FSHD patients and 29 healthy controls. Fifty samples (39 patients and 11 controls) were collected at RUMC, whereas the remaining 33 samples (15 patients and 18 controls) were collected at UNEW (Table 1). For FSHD patients, the median FSHD clinical score^28^ was 7 at RUMC and 11 at UNEW, indicating that the patients followed at UNEW typically had a more advanced stage of the disease. The study was approved by the institutional review boards of the involved clinical centres. Informed consent forms were obtained for all participants. The investigation was conducted according to the declaration of Helsinki.

**Table 1:** Overview of the subjects included in this study.

	RUMC	UNEW
**Number of subjects (of which: males)**	50 (27)	33 (9)
FSHD patients	39 (23)	15 (9)
Healthy controls	11 (4)	18 (0)
**Age: median (range)**	52 (40; 60)	45 (24; 71)
FSHD patients	51 (40; 60)	49 (24; 71)
Healthy controls	54 (42; 60)	42 (27; 61)
**FSHD clinical score: median (range)**		
FSHD patients	7 (0; 15)	11 (2; 15)
Healthy controls	NA	NA

A total of 83 individuals were included in the study, 50 from the RUMC cohort and 33 from the UNEW cohort. The table reports the number of patients by cohort, group and gender; the median and range of age by cohort and group; and the median FSHD score and its range by cohort for FSHD patients (*NA* not applicable).

### RNA sequencing

Blood samples were collected in PAXgene tubes and stored at − 80 °C prior to use. RNA was purified and depleted for globin mRNA content using the globin clear kit (cat AM1980, Ambion—ThermoFisher). A qPCR assay was performed to assess the expression of globin mRNA before and after mRNA depletion. RNA quality was assessed using the Agilent bioanalyzer. The Illumina TruSeq RNA Library Prep Kit v2 was used to process the samples. Sample preparation was assessed by a Fragment Analyzer, and the distribution size was consistent with a broad peak of 300–500 bp. Paired end sequencing was performed. Sample preparation was performed separately for the 2 cohorts.

All RNA sequence files were processed using the BIOPET Gentrap pipeline version 0.9 developed at the LUMC (https://github.com/biopet/biopet), previously described in Ünlü et al.^29^. BIOPET Gentrap pipeline consists of FASTQ preprocessing (including quality control, quality trimming and adapter clipping), RNA-seq alignment, read and base quantification, and optionally transcript assembly. FastQC version 0.11.2 was used for raw read quality control. Low quality read trimming was done using sickle version 1.33 with default settings. Cutadapt version 1.10 with default settings was used for adapter clipping based on the detected adapter sequences by FastQC toolkit. RNA-seq reads were aligned against human reference genome GRCh38 without alternative contigs using RNA-seq aligner GSNAP version 2017–09-11, with settings "–npaths 1" to allow multimapped reads to be aligned to one location. Ensembl gene annotation version 87 was used for raw read counting. This gene read quantification step was performed using htseq-count version 0.6.1p1 with settings of "–stranded = no".

The expression profile of all repeat elements defined in the RepeatMasker track (accessed on June 20, 2019) from the UCSC genome browser was generated using featureCounts, with options “-M -O –fraction” to allow fractional counting for overlapping features and multimapped reads.

Alignment with the *DUX4* locus was further investigated using LAST aligner, which enables the alignment to efficiently cope with repeat-rich sequences. *GAPDH* and *ACTB* were taken along as control loci. The 4qA35 sequence, always found in FSHD patients, was used as a reference sequence as it includes two D4Z4 repeat units, the *DUX4* open reading frame and the pLAM region including the polymorphic *DUX4* polyadenylation site. Alignment with lastal command was done with the seeding scheme NEAR. The initial match or seed with this scheme was 7 bases in length and could only contain 1 mismatch per seed, which created strong initial matches. NEAR also determined the scoring of the mapped reads by setting the match score at 6, the mismatch cost at 18, the gap cost at 21, and the gap extension cost at 9. To align read fragments of more than 50 bp in length and allow some mismatches, the minimum alignment score was fixed at 200. The maximum multiplicity was increased to 50, so more initial matches were taken into account into the scoring. Lastal produced a maf document with the mapped reads and their scores, which was converted to a sam document with maf-convert.

### Filtering, normalization and estimation of cell types proportions

In order to remove lowly expressed genes from our analyses, we filtered out genes with less than 5 counts per million (cpm) in more than 90% of the samples in the discovery cohort (RUMC). However, considering that genes in the *DUX4* and *PAX7* signatures^6,21^ have been previously reported to discriminate FSHD patients from controls, we retained genes belonging to these two signatures even when they did not satisfy the filtering criteria provided that they were expressed in at least 1 sample in each cohort. None of the genes belonging to the *DUX4* signature passed the filtering threshold, but 57 were manually included in the analysis. A total of 338 genes belonging to the *PAX7* signature passed the filtering step, and a further 336 were manually included. The total number of genes considered in the analysis was 11,854. Estimation of the proportion of different blood cell types (neutrophils, lymphocytes, monocytes, basophils and eosinophils) was performed using the R package wbccPredictor^30^. RNA-seq expression profiles were normalized separately in each cohort using the Trimmed Mean of M values normalization method^31^.

### Differential expression tests

In order to identify genes differentially expressed between FSHD patients and controls, we considered negative binomial generalized linear models (GLMs) where the logarithm of the expected value of each gene depends on group, age, gender and on the estimated proportion of neutrophils, monocytes and eosinophils. Such models were fitted using the R package edgeR^32^. The percentages of lymphocytes and basophils were not included as covariates because the percentages of lymphocytes and neutrophils were found to be multicollinear (Pearson’s correlation coefficient = − 0.94 for the RUMC and − 0.92 for the UNEW cohort), and the percentage of basophils was estimated to be negligible (< 1.2%) in all samples (Supplementary Fig. S1). We employed a quasi-likelihood F test to test the significance of group differences. P-values were adjusted for multiple testing using the Benjamini-Hochberg (BH) method^33^.

Differential expression at the pathway level was tested using the globaltest^34^, considering as pathways the 2,255 Reactome pathways, as well as the *DUX4* and *PAX7* signatures. We retained for analysis all the pathways (2,074 Reactome pathways, *DUX4* signature and *PAX7* signature) that contained at least one of the 11,854 genes considered in our analysis. For each cohort, gene expression levels were converted to log-normalized counts per million before inclusion as covariates in the globaltest. We accounted for the same confounders (age, gender and proportion of neutrophils, monocytes and eosinophils) used in the gene-wise tests. Multiple testing correction was implemented using the BH method.

Lastly, we tested whether the distribution of the PAX7 score and the three DUX4 scores proposed by Banerji and Zammit^21^ differed between the FSHD and healthy groups. We computed both the Wilcoxon test employed in the original publication, which does not allow to correct for relevant confounders, and the P-value for group differences obtained from a linear regression model where, besides modelling group differences, we control for age, gender and for the estimated proportion of neutrophils, monocytes and eosinophils. P-values were corrected for multiple testing using the BH method.

### Network enrichment analyses

Network enrichment analysis was performed considering all gene–gene interactions present in Funcoup version 4^35^ and using the NEAT^36^ test. Because the power to detect network enrichment is typically low for small gene sets, we only considered those Reactome pathways that had at least 5 genes present in Funcoup (1,765 out of 2,255), as well as the *DUX4* and *PAX7* signatures. P-values were adjusted for multiple testing using the BH method^33^. Significant network enrichments were classified as “over-enrichments” if the observed number of links between a pathway and the gene set of interest was bigger than that expected in absence of enrichment, and as “under-enrichments” if lower than expected.

### Evaluation of the predictive performance of different gene signatures

We employed logistic regression to quantify how well different gene signatures could predict whether an individual suffers from FSHD. For this analysis, we combined samples from RUMC and UNEW after having converted the RNA-seq measurements to normalized counts per million. In each logistic model we always included gender and age as predictors, and added either a single gene or all the genes belonging to a given gene set. Models with at most 3 covariates were estimated with maximum likelihood, whereas models comprising 8 or more covariates were estimated with penalized maximum likelihood, considering a logistic model with ridge penalty and selecting the tuning parameter by cross-validation. We employed the area under the ROC curve (AUC) to quantify the predictive accuracy of each model. Split-sample validation, stratified by group, was performed by allocating 70% of the subjects to the training set and the remaining 30% to the validation set. The validation procedure was repeated 100 times for each model, and the distribution of AUC in the validation set across repetitions was compared across models using boxplots.

### Identification of genes associated with the FSHD clinical score

With the aim of identifying genes associated with the FSHD clinical score^28^, we considered only samples from FSHD patients and employed edgeR to fit negative binomial GLMs where the logarithm of the expected value of each gene depends on the clinical score, gender, age and on the estimated proportion of neutrophils, monocytes and eosinophils. We employed a quasi-likelihood F test to test the significance of the regression coefficient associated to the FSHD clinical score. P-values were adjusted for multiple testing using the BH method.

### Analysis of repeated elements

We tested whether any repeated element was differentially expressed between FSHD patients and controls using negative binomial GLMs where the logarithm of the expected value of each repeated element depends on group, age and gender. Such models were fitted using edgeR^32^. For this analysis we only considered repeated elements that were expressed in at least 80% of the samples. We employed a quasi-likelihood F test to test the significance of group differences. P-values were adjusted for multiple testing using the BH method^33^.

## Results

### Characterization of differences in gene expression of FSHD patients

PAXgene tubes were obtained from 54 FSHD1 patients and 29 healthy controls across two independent cohorts (Table 1). Sequencing of RNA obtained from PAXgene tubes resulted in an overall good alignment, with a percentage of aligned reads that ranged between 65.7% and 78.8% across samples. The total number of aligned reads was highly variable across samples, ranging from 10.4 to 90 million reads, and it was typically higher in samples from the UNEW cohort (43.8 million reads on average) than in those from the RUMC cohort (17.5 million reads on average) due to deeper sequencing of the UNEW cohort.

No reads aligned to the *DUX4* gene using GSNAP alignment to the GRCh38 build. To further assess whether alignment to the locus was affected by the sequence context and repeated sequences, a second aligner better suited for repeated sequences was used; however, again no reads were found to align to the unique somatic polyadenylation site of *DUX4*.

**Figure 1:**
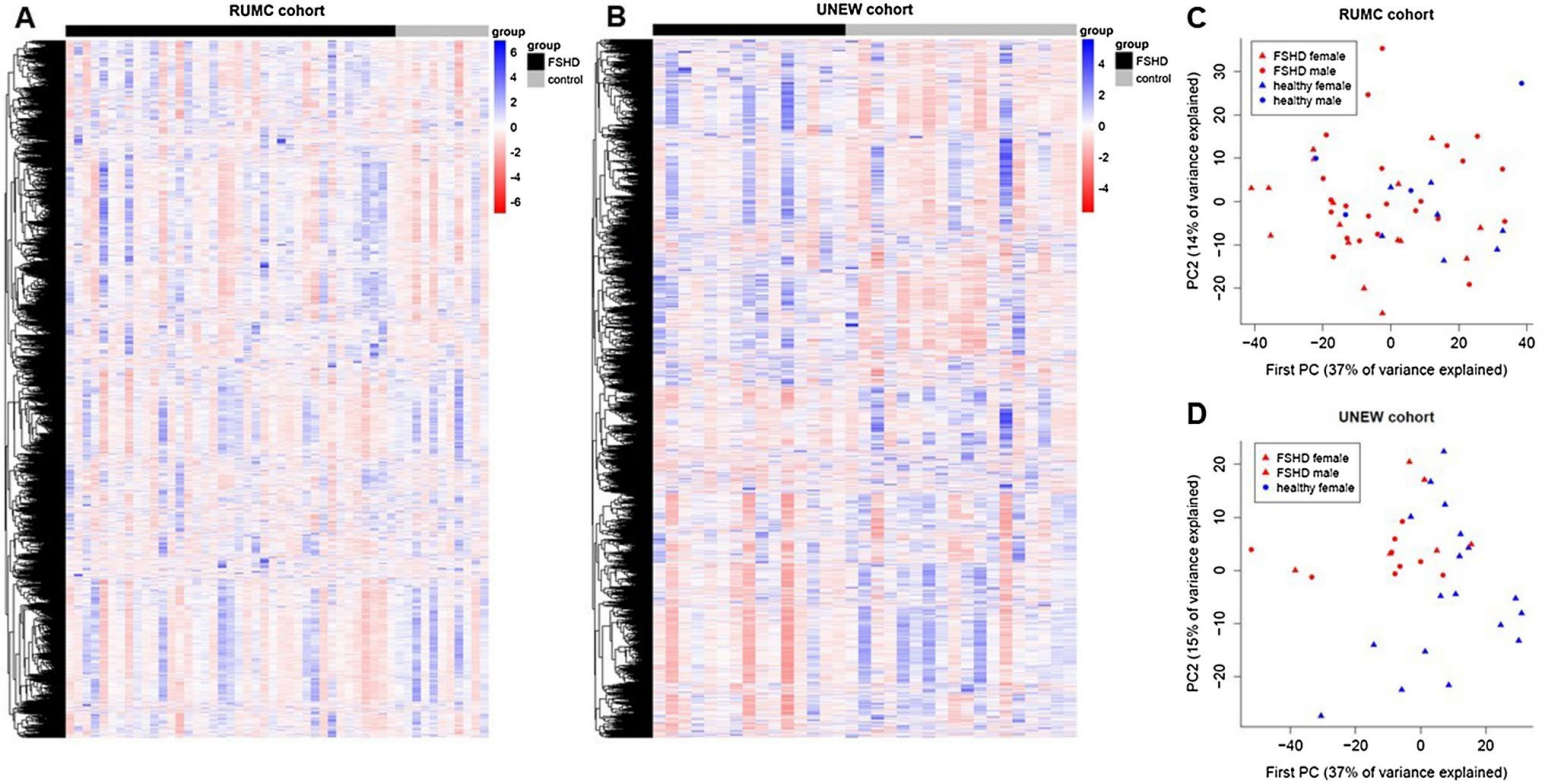
Exploratory analysis of RNA-seq data. (**A**,**B**) Heatmaps comparing gene expression between FSHD patients and healthy individuals in the RUMC (**A**) and UNEW (**B**) cohorts. On the x axis, samples are ordered by group. On the y axis, genes are clustered to display genes with similar expression patterns next to each other. (**C**,**D**) Scatterplots with the values of the first and second principal component in the RUMC (**C**) and in the UNEW (**D**) cohorts. FSHD patients and healthy individuals are denoted by red and blue triangles, respectively. The proportion of variance explained by each component is reported on the corresponding axis.

Aligned reads were used to generate heatmaps to visualize the distribution of normalized RNA-seq counts across samples; comparison of gene expression in FSHD and healthy controls showed smaller differences in the RUMC cohort (Fig. 1A), while a stronger signal was present in the UNEW cohort (Fig. 1B). We further employed principal component analysis to project the samples onto a low-dimensional space defined by the first two principal components (PCs). The percentage of variance explained by the first two PCs was very similar across cohorts (37% for PC1, 14–15% for PC2). Graphical inspection of the PC scores showed a substantial overlap between FSHD cases and healthy controls in the RUMC cohort (Fig. 1C), while the two groups formed somewhat separate clusters in the UNEW cohort (Fig. 1D).

**Figure 2:**
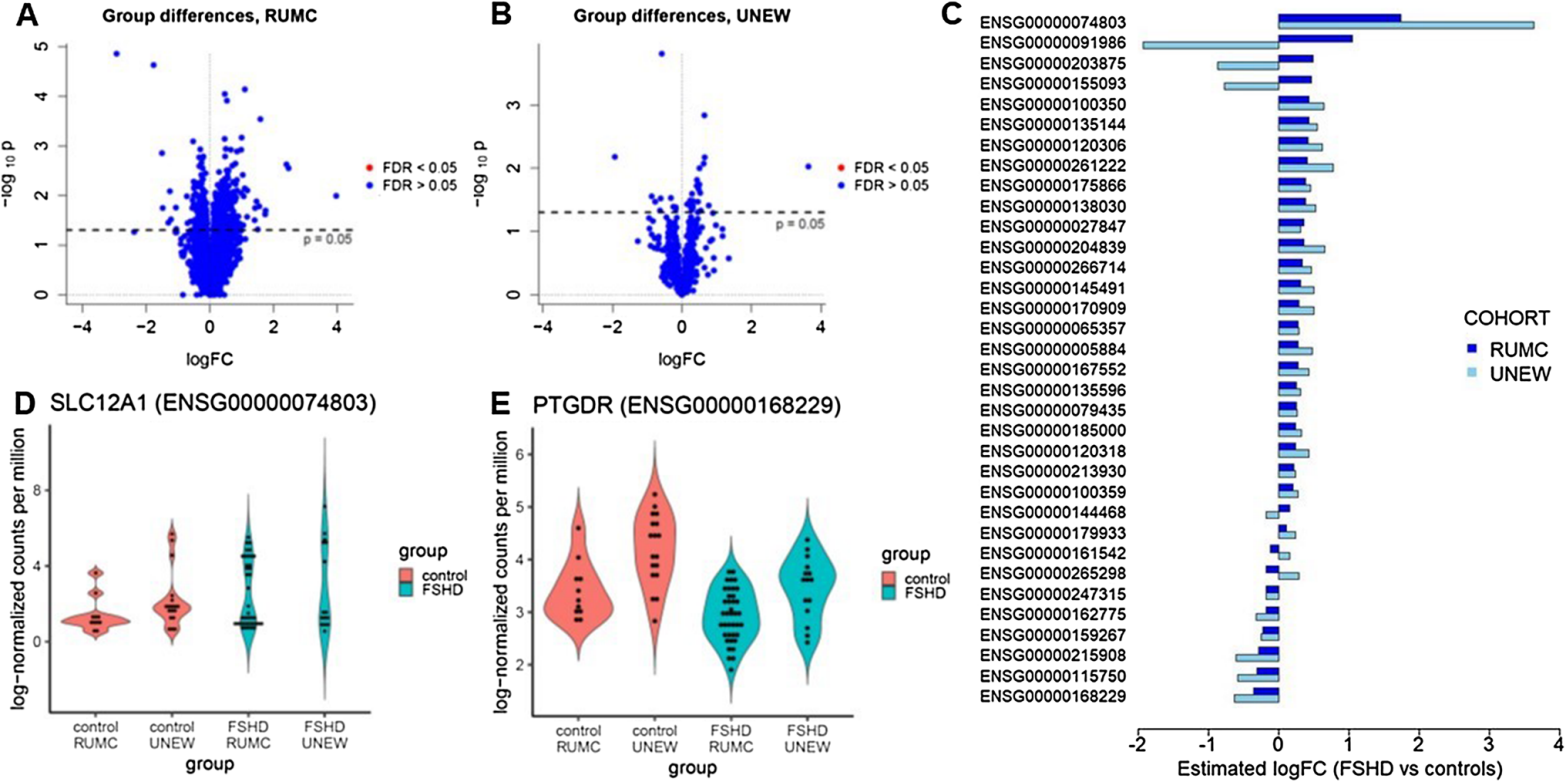
Tests for differential expression between controls and FSHD patients. (**A**,**B**) Volcano plots for the tests of differential expression in the RUMC (**A**) and in the UNEW (**B**) cohorts. (**C**) Barplot comparing the estimated logFCs in the RUMC and UNEW cohorts for the 30 genes with p < 0.05 in both cohorts. (**D**,**E**) Violin plots comparing the distribution of the log-normalized counts per million of *SLC12A1* (ENSG00000074803, **D**) and *PTGDR* (ENSG00000168229, **E**) in FSHD patients and healthy controls.

We tested differential expression between FSHD patients and healthy controls using negative binomial GLMs where we controlled for gender, age and the cell type proportions estimated with wbccPredictor (Supplementary Fig. S1) as possible confounders. We selected the largest cohort from RUMC as discovery cohort and used the UNEW cohort for validation purposes. We identified 764 genes with a p-value < 0.05 in the RUMC cohort, but no difference was found to be significant (FDR > 0.05 for all genes) after multiple testing correction (Fig. 2A and Supplementary File 1). Among the 764 genes, 31 belonged to the *PAX7* signature, while none of the *DUX4* target genes were present. The 764 genes with p < 0.05 in the RUMC cohort were further tested in the UNEW cohort. Although a p-value < 0.05 was observed for 34 genes in the second cohort, none of the estimated differences was significant after multiple testing correction (Fig. 2B and Supplementary File 2).

To evaluate the level of agreement of these results across both cohorts, we compared the magnitude and direction of the estimated log-fold changes (logFCs) for the 34 genes with p < 0.05 in both cohorts. Interestingly, for 82% of the genes (28 out of 34) the estimated logFCs showed concordant signal (Fig. 2C and Supplementary File 3), indicating that the direction of differences between the two groups are typically consistent across cohorts. No gene belonging to the *PAX7* signature was present among the 28 genes with concordant FC direction. *SLC12A1* (ENSG00000074803) was the gene with the largest logFCs (1.74 in the RUMC and 3.63 in the UNEW cohort), and it was found to be elevated in FSHD patients (Fig. 2D); on the other hand, *PTGDR* (ENSG00000168229) was the gene with the strongest negative logFCs (-0.36 in the RUMC and -0.63 in the UNEW cohort; Fig. 2E). We further employed network enrichment analysis to assess the connectivity of those 28 genes (referred to as altered gene set 1, or AGS1, hereafter) in the Funcoup gene interaction network. Twenty-five of the 28 AGS1 genes were present in Funcoup. The number of their connections in the network was highly variable (Supplementary Fig. S2A), ranging from 1 for *MYO15B* (ENSG00000266714) to 4,192 for *TUBA1A* (ENSG00000167552), giving an indication that while some genes in the list occupy a central position in the network, others are in a more peripheral position. To characterize the connections that genes in AGS1 have to the rest of the network, we tested network enrichment of AGS1 against the Reactome pathways and the *DUX4* and *PAX7* signatures. We identified 245 pathways (Supplementary Fig. S2B) as network-enriched, i.e. as pathways with a number of connections with AGS1 that is significantly different from the number of connections expected in absence of network enrichment. Among these 245 significant pathways, of particular interest are 191 pathways that are over-enriched, i.e. they are significantly more connected to AGS1 genes than it would be expected in absence of network enrichment (Supplementary File 4). Metabolic pathways showed the strongest network enrichment. Both the *DUX4* and the *PAX7* gene sets did not show enrichment in connection to AGS1.

In addition to testing differential expression of individual genes, we also tested differential expression at the pathway level using the global test^34^. We tested differential expression of 2,076 pathways (2,074 Reactome pathways plus the *DUX4* and *PAX7* signatures) in the RUMC cohort, but did not find any pathway to be significantly different after correcting for multiple testing (Supplementary Fig. S3A). We also tested the 97 pathways for which p < 0.05 in the RUMC cohort in the UNEW cohort, but did not find any of those to be significantly different after multiple testing correction (Supplementary Fig. S3B). Lastly, we computed the *PAX7* and *DUX4* scores proposed by Banerji and Zammit^21^. In the RUMC cohort none of the four scores displayed significant differences, irrespective of whether confounders are ignored or accounted for in the testing procedure. In the UNEW cohort, the *PAX7* score and one of the *DUX4* scores were found to differ between FSHD and healthy patients when looking at the unadjusted difference between groups; however, the difference was not significant after accounting for confounders such as gender, age and cell type proportions (Supplementary File 5).

### Classification based on individual genes and gene sets

To assess the potential of RNA-seq blood data to improve classification of patients and healthy individuals, we compared the area under the ROC curve of different logistic models. We first considered a model where only information on gender and age was included; this model achieved a mean AUC of 72.9% (95% confidence interval: 46.4–89.7%). This value indicates that it is possible to achieve an AUC of 72.9% in the classification of the samples collected in this study by only using information on gender and age.

**Figure 3:**
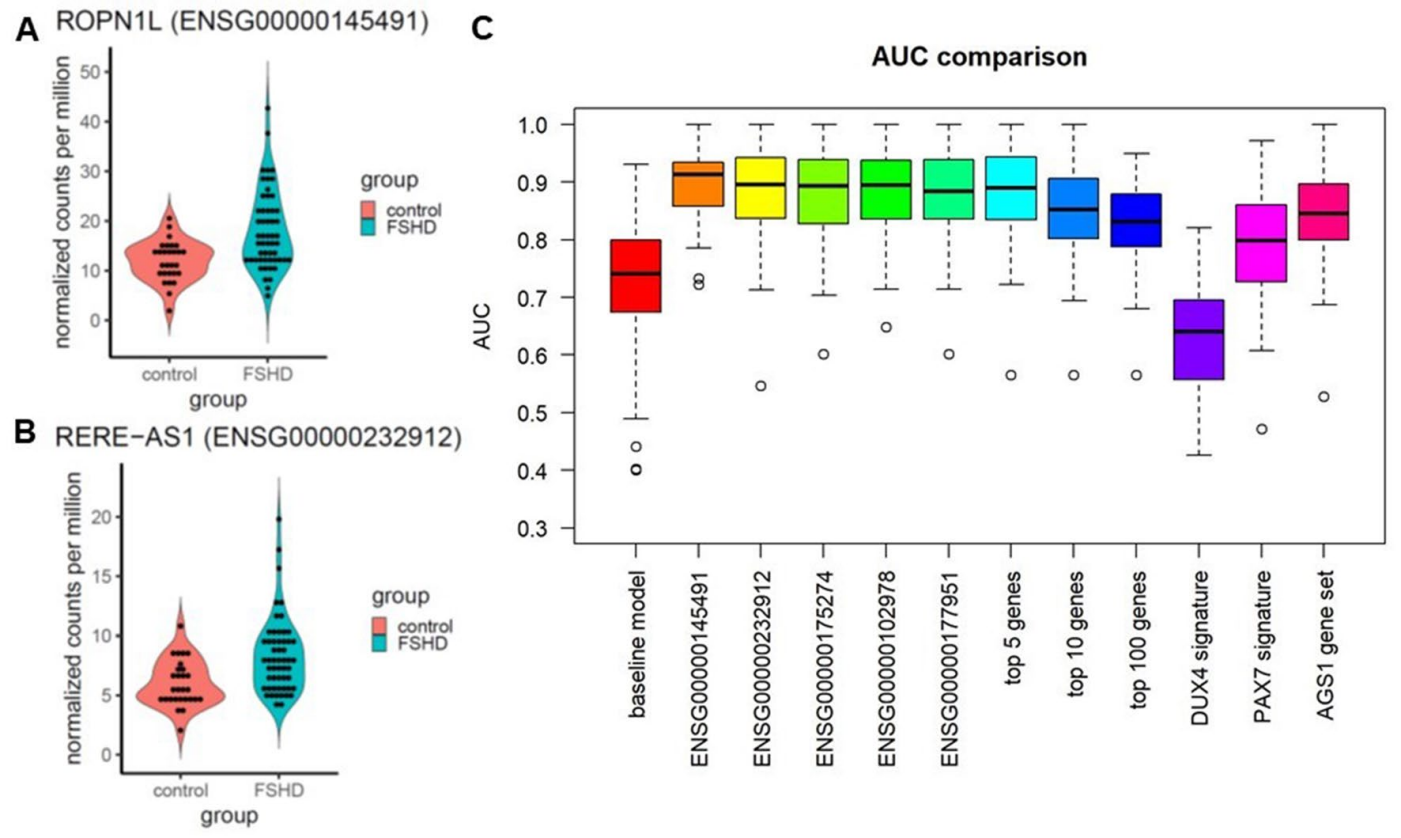
Predictiveness of individual genes and gene signatures. (**A**,**B**) Violin plots comparing the distribution of *ROPN1L* (ENSG00000145491, **A**) and *RERE-AS1* (ENSG00000232912, **B**) in the FSHD and control groups. (**C**) Boxplots comparing the distribution of the AUC of selected models over 100 split-sample validation replicates. The boxplots display the AUC distribution of: the baseline model (age and gender as predictors); the five models with highest mean AUC among those with age, gender and one single gene as predictors; three models with age, gender, and the top 5, 10, or 100 genes as predictors; two models with age, gender, and genes from the DUX4 or PAX7 signature as predictors; and a model with age, gender, and the genes from the AGS1 gene set.

We then assessed how the expression levels of a single gene can improve classification, adding to age and gender each of the 11,854 genes considered in our study. In Table 2 we report the top 10 models with the highest AUC; detailed results for all genes are given in Supplementary File 6. The gene that improved classification the most was *ROPN1L* (ENSG00000145491), achieving an AUC of 89.8%, 16.9% higher than that of the baseline model with only age and gender (Fig. 3A). This gene is also among the 28 AGS1 genes with p < 0.05 and concordant sign across cohorts (Fig. 2C), and it is on average elevated in FSHD patients. *RERE-AS1* (ENSG00000232912), often elevated in FSHD patients (Fig. 3B), resulted in a mean AUC of 88.5%, improving classification by 15.7%. Out of the 28 genes in AGS1, 23 achieved an AUC of at least 80% (Supplementary File 6).

**Table 2:** Top 10 genes by predictive accuracy.

Gene	Mean AUC	95% confidence interval	AUC improvement
ROPN1L (ENSG00000145491)	89.8%	(79.2%; 99.6%)	16.9%
RERE-AS1 (ENSG00000232912)	88.5%	(71.8%; 100%)	15.7%
TP53I11 (ENSG00000175274)	88.2%	(76%; 100%)	15.3%
POLR2C (ENSG00000102978)	88.2%	(72.6%; 99.2%)	15.3%
BET1L (ENSG00000177951)	88.1%	(73.6%; 99.6%)	15.2%
ZBTB7B (ENSG00000160685)	88.1%	(74.4%; 99.2%)	15.2%
CEACAM3 (ENSG00000170956)	88%	(74.8%; 99.4%)	15.2%
OSCAR (ENSG00000170909)	88%	(73.4%; 100%)	15.2%
ZDHHC3 (ENSG00000163812)	87.9%	(73.9%; 99.6%)	15.1%
TFE3 (ENSG00000068323)	87.9%	(76%; 99.1%)	15%

This table lists the 10 genes that, when added to gender and age, increase prediction accuracy the most (mean AUC using only age and gender = 72.9%).

We also checked whether selected combinations of genes could further improve classification by adding to gender and age five different sets of genes as covariates (Fig. 3C). Use of the top 5 genes in Table 2 resulted in a mean AUC of 88.3% (95% CI 73.5–100%), achieving a discriminatory performance comparable to that of those genes when used individually. Adding further genes deteriorated the classification performance (mean AUC using the top 10 genes: 85.3%; using the top 100 genes: 82.9%). Use of genes from the *DUX4* signature as classifiers resulted in a poor AUC (mean AUC 63%, 95% CI: 46.2–77.9%), and also use of the *PAX7* signature resulted in an AUC considerably lower than that of the genes in Table 2 (mean AUC 79.4%, 95% CI 61.8–96.1%). Use of the AGS1 genes produced a mean AUC of 84.5% (CI 71.3–97.5%).

### Assessing the association between gene expression and the FSHD clinical score

**Figure 4:**
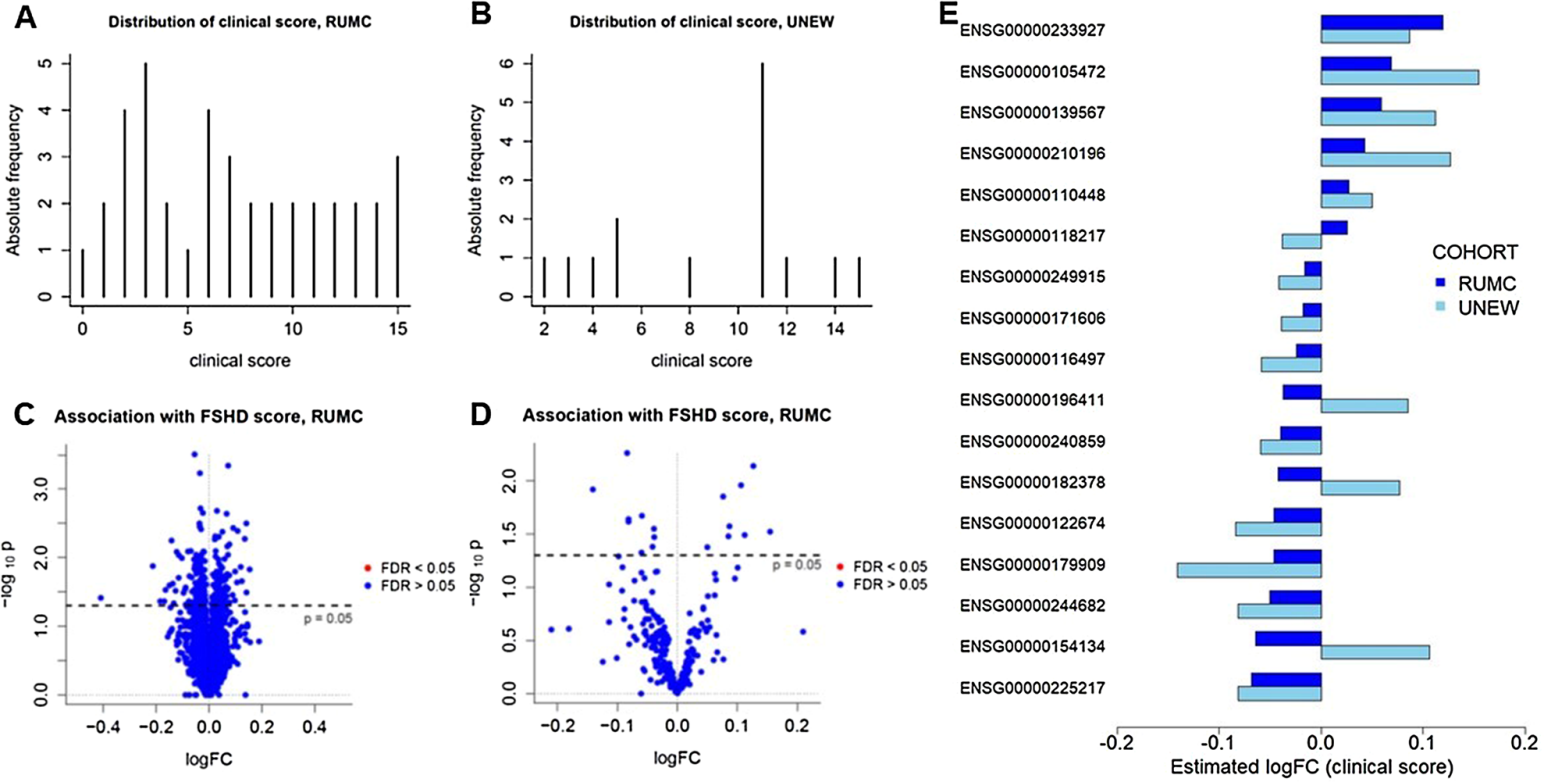
Tests on the association of individual genes with the FSHD clinical score. (**A**,**B**) Distribution of the FSHD clinical score in the RUMC (**A**) and UNEW (**B**) cohorts. (**C**,**D**) Volcano plots for the association tests between clinical score and gene expression in the RUMC (**C**) and in the UNEW (**D**) cohorts. (**E**) Barplot comparing the estimated logFCs in the RUMC and UNEW cohorts for the 17 genes with p < 0.05 in both cohorts.

To assess whether a connection exists between blood gene expression and clinical performance, we investigated the association between gene expression and the FSHD clinical score^28^, a discrete score ranging between 0 and 15 that measures the severity of muscle weakness in six body regions of FSHD patients (Fig. 4A,B; note that higher scores indicate higher severity). For each gene we tested the null hypothesis that its average expression level is not associated with the clinical score after correcting for possible confounders such as gender, age, and the estimated cell type percentages. We selected the largest cohort from RUMC as discovery cohort, and tested the significance of the association for all genes. We identified 248 genes with a p-value < 0.05 in the RUMC cohort, but no difference was significant (FDR > 0.05 for all genes) after multiple testing correction (Fig. 4C and Supplementary File 7). Among the 248 genes, 32 belonged to the *PAX7* signature and one (*ALPG* / ENSG00000163286) to the *DUX4* signature.

The 248 genes with p < 0.05 in the RUMC cohort were further tested in the UNEW cohort. Although a p-value < 0.05 was observed for 17 out of the 248 genes in the second cohort, none of the estimated associations were significant after multiple testing correction (Fig. 4D and Supplementary File 8). However, for 13 of these 17 genes the sign of the estimated logFCs was  concordant across cohorts (Fig. 4E and Supplementary File 9), indicating that for the top ranking genes the sign of associations with the clinical score was usually consistent across cohorts. One of the 13 genes, *ACVRL1* (ENSG00000139567), is part of the PAX7 signature.

We further employed network enrichment analysis to assess the connectivity of those 13 genes (referred to as altered gene set 2, or AGS2, hereafter) in Funcoup. Ten of the 13 AGS2 genes are present in Funcoup, and they have a very heterogeneous number of links to other genes in the network (Supplementary Fig. S4A), ranging from 6 to 3682. To characterize the connections that genes in AGS2 have to the rest of the gene interaction network, we tested network enrichment of AGS2 against the Reactome pathways and the *DUX4* and *PAX7* signatures. We identified 62 enriched pathways (Supplementary Fig. S4B), of which 29 are over-enriched (Supplementary File 10); the *DUX4* and the *PAX7* gene sets were not network-enriched with AGS2. Interestingly, AGS2 genes appear to be highly connected to genes involved in the complement activation pathway, a pathway previously reported to be over-enriched in gene expression data obtained from FSHD muscle biopsies^37^.

### Analysis of repeated elements

**Figure 5:**
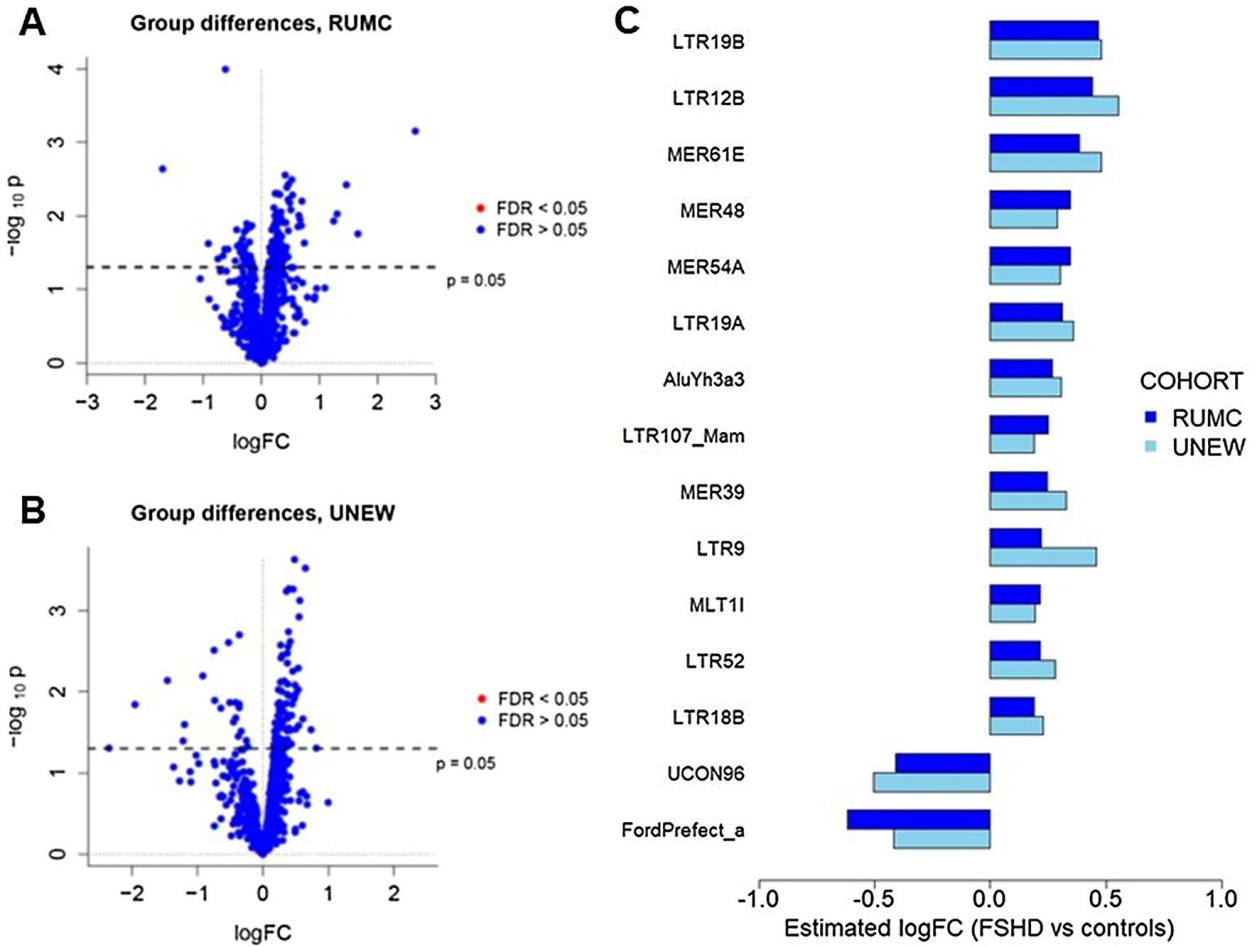
Analysis of repeated elements. (**A**,**B**) Volcano plots for the tests of differential expression between FSHD patients and controls in the RUMC (**A**) and in the UNEW (**B**) cohorts. (**C**) Barplot comparing the estimated logFCs in the RUMC and UNEW cohorts for the 15 repeated elements with p < 0.05 in both cohorts.

Since DUX4 has been reported to affect the expression of repeated elements^38^, we tested whether repeated elements in the genome were differentially expressed in the blood expression data of FSHD patients compared to healthy controls. The expression of all repeated elements present in repeat masker were considered for this analysis. We identified 151 elements with p < 0.05 in the RUMC cohort (Fig. 5A and Supplementary File 11) and 110 elements with p < 0.05 in the UNEW cohort (Fig. 5B and Supplementary File 12); however, none of the identified differences were significant after correcting for multiple testing (DSR > 0.05 for all elements in both cohorts). Next, we examined the level of agreement across cohorts, comparing the magnitude and direction of the estimated log-fold changes (logFCs) for the 15 elements with p < 0.05 in both cohorts. Overall, all of the estimated logFCs were rather small, but their sign was concordant across cohorts for all 15 elements (Fig. 5C and Supplementary File 13).

## Discussion

FSHD is caused by the de-repression of the chromosome 4 D4Z4 repeat array, which results in the expression of *DUX4* in skeletal muscle. This leads to a complex cascade of events resulting in a dystrophic phenotype. Analysis of gene expression and epigenetic data from muscle cell cultures have enabled to decipher the events preceding and following *DUX4* expression^15,23,38,39^ clarifying the role of *DUX4* in FSHD pathophysiology. While evidence is still being collected, testing and development of drugs aiming to antagonize the expression of *DUX4*, its upstream regulators and downstream events is currently ongoing, with both planned and running clinical trials. Given the slow progressive nature of FSHD, with variable disease trajectories and phenotype improvements over a short period^24^, initiatives were taken to develop a clinical trial toolkit for use when evaluating responses to drugs. The identification of biomarkers connected to the *DUX4* signature and associated with clinical performance are therefore needed to inform clinical trial design and allow interpretation of the outcomes.

In this work we studied gene expression in peripheral blood of FSHD patients and healthy controls from 2 independent cohorts. RNA sequencing was used with the intention to look for differentially expressed genes, test whether gene expression can improve the classification of patients and controls, and identify associations with clinical performance.

The expression of *DUX4* was not detectable in blood, proving that the detection (and quantification) of the DUX4 transcript as diagnostic and possibly PD biomarker will be challenging. While this was expected given the low and burst-like expression of the *DUX4* gene in muscle, downstream genes have been shown to be detectable targets in muscle tissue and cultured cells^6^. We therefore included the *DUX4* signature in the analysis, even though all genes were below the filtering threshold (less than 5 cpm in more than 90% of the samples). In addition we included *PAX7* regulated genes, as it was recently suggested that the repression of these genes represent a superior biomarker compared to the *DUX4* signature^21^.

Gene expression data of FSHD patients and healthy controls largely overlapped, and no gene was identified as differentially expressed between the two groups after multiple testing correction. Pathway analysis further confirmed that no significant differences existed in the blood expression profiles between FSHD and healthy individuals. While it is possible that no gene is indeed differentially expressed, the inability to detect significant associations could be due to the small sample size and lack of follow-up samples, e.g. samples from the same individual following disease progression. The number of patients involved in the study is indeed quite small when compared to other diseases where hundreds of patients are recruited^40^. The results of our study are similar to those of comparable studies involving a comparable number of patients in disease areas such as cluster headache and Huntington’s disease^41,42^, which reported that little to no change was found in blood gene expression by RNA-seq. The difficulty to identify differentially expressed genes in such studies may be due to a number of factors such as the small effect sizes observed in blood for these diseases, small sample sizes, and the choice of RNA-seq as sequencing technology. Indeed, while analysis by SAGE-seq successfully identified differentially expressed genes in blood of patients affected by Huntington’s disease^43^, RNA-seq failed to show significant changes^42^.

Despite the lack of significant associations, evidence suggests that the analysis of blood-derived RNA could provide potential biomarkers for FSHD. A number of genes showed a nominal p-value < 0.05 in both cohorts along with a concordant directional change. Classification of cases and controls was largely improved by RNA-seq data, up to an AUC of 89.8%, a figure comparable with the 87% AUC reported for patients affected by amyotrophic lateral sclerosis in a much larger cohort composed of hundreds of cases^40^. An improvement of ≥ 15% in case and control classification was achieved by adding a single gene to the model, showing that targeted approaches focusing on the identified classifiers would be highly feasible for independent cohorts. The gene showing largest improvement in classification was *ROPN1L*, a gene mainly expressed in testes. Interestingly, the testis are where *DUX4* is normally expressed and activates the transcription of retro-elements among other genes^39,44^. We therefore assessed whether all repetitive elements listed in repeat masker were differentially expressed in blood from FSHD individuals. 15 elements had a nominal p-value < 0.05 and concordant directional change, but results were not significant after correcting for multiple testing. This suggests that there may be a potential repeated elements signature, multiple of which were indeed LTRs, in FSHD patient blood.

Among the top 10 classifiers, we identified *TFE3*, a broadly expressed transcription factor linked to cytokine expression^45^, SMAD3 signaling in response to TGF-β^46^ and CD40 expression^47^, recently shown to be a genetic modifier of muscular dystrophy^48^. Future studies should aim to understand the connection between *DUX4* and the inflammatory—immune response, where transcription factors such as *TFE3* play an important role.

Our data show that the expression of genes belonging to the *DUX4* signature is low in blood-derived samples, suggesting that using the *DUX4* signature in blood as PD readout for *DUX4* antagonizing therapies will be challenging. Both *DUX4* and *PAX7* signatures were outperformed in classification analysis by the AGS1 gene-set identified in this study. Further studies should aim to assess whether the expression levels of genes belonging to the identified gene-set in blood correlate to the expression in muscle tissue to define the context of use for these potential biomarkers.

Lastly, no genes were found to be associated with patients’ disease severity as measured by the FSHD clinical score. While it is possible that no real association exists, the identification of genes associated with a composite clinical score could complicate such analysis. Prospective studies should aim to identify blood-based biomarkers that are able to predict the likelihood of a clinically relevant event for FSHD patients. Identification of such associations would be easier to interpret and likely more clinically relevant.

Our study presents a number of limitations such as the number of patients involved, the lack of longitudinal follow-up samples, the presence of only female healthy controls in the UNEW cohort and the lack of information on disease activity by MRI (STIR positivity). Despite these limitations, the study provides a comprehensive analysis of potential candidate biomarkers that could be included in future studies. If confirmed by future studies, such candidates could enrich the FSHD clinical trial toolbox.

## Supplementary information

Supplementary Figures.

Supplementary Information 1.

Supplementary Figure legends.
