# Supplementary material for: Evaluation of blood gene expression levels in facioscapulohumeral muscular dystrophy patients
Source: Sci Rep. 2020 Oct 16;10:17547. doi: 10.1038/s41598-020-74687-5 (PMC7567883; doi:10.1038/s41598-020-74687-5)
Supplement: Supplementary file 1 — Supplementary Figures. [file 41598_2020_74687_MOESM1_ESM.pdf]

Supplementary figures of the manuscript "Evaluation of blood gene expression levels in facioscapulohumeral muscular dystrophy patients"

Authors

M. Signorelli[1,*], A.G. Mason[2,*], K. Mul[3], T. Evangelista[4,5], H. Mei[1], N. Voermans[3], S.J. Tapscott[6,7], R. Tsonaka[1], B.G.M. van Engelen[3], S.M. van der Maarel[2], P. Spitali[2]

Affiliations

1. Department of Biomedical Data Sciences, Leiden University Medical Center, 2333 ZC, Leiden, The Netherlands

2. Human Genetics Department, Leiden University Medical Center, 2333 ZC, Leiden, The Netherlands

3. Department of Neurology, Donders Institute for Brain, Cognition and Behaviour, Radboud University Medical Center, Nijmegen, The Netherlands

4. John Walton Muscular Dystrophy Research Centre, Institute of Genetic Medicine, Newcastle University, Centre for Life, Newcastle, NE1 3BZ, UK.

5. Sorbonne Université, AP-HP, INSERM, Centre de référence des maladies neuromusculaires Nord/Est/Ile de France, Groupe Hospitalier Universitaire La Pitié-Salpêtrière, Paris, France6

6. Human Biology Division, Fred Hutchinson Cancer Research Center, Seattle, WA 98109, USA

7. Department of Neurology, University of Washington, Seattle, WA 98105, USA

*Equal contributors

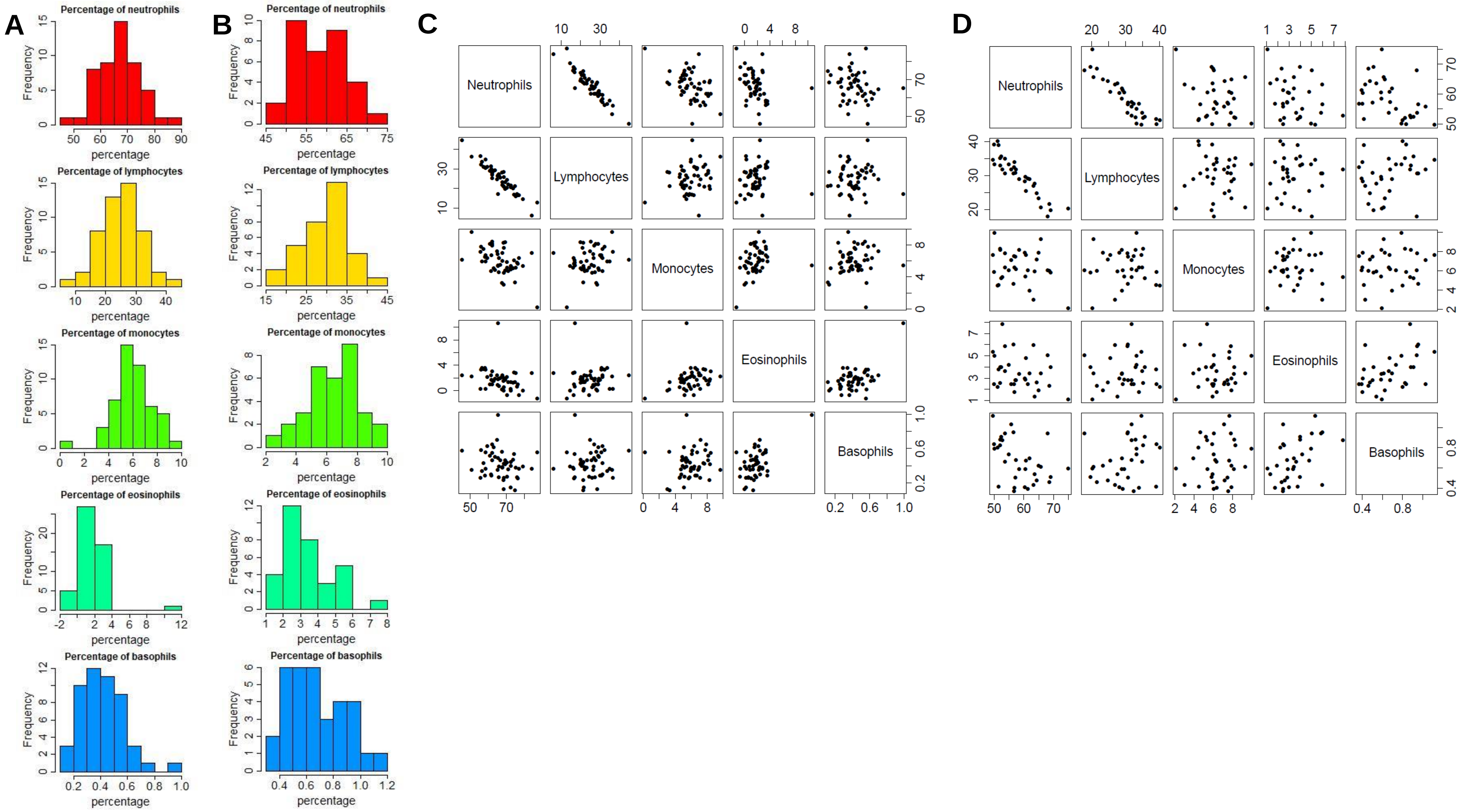

Supplementary Figure 1

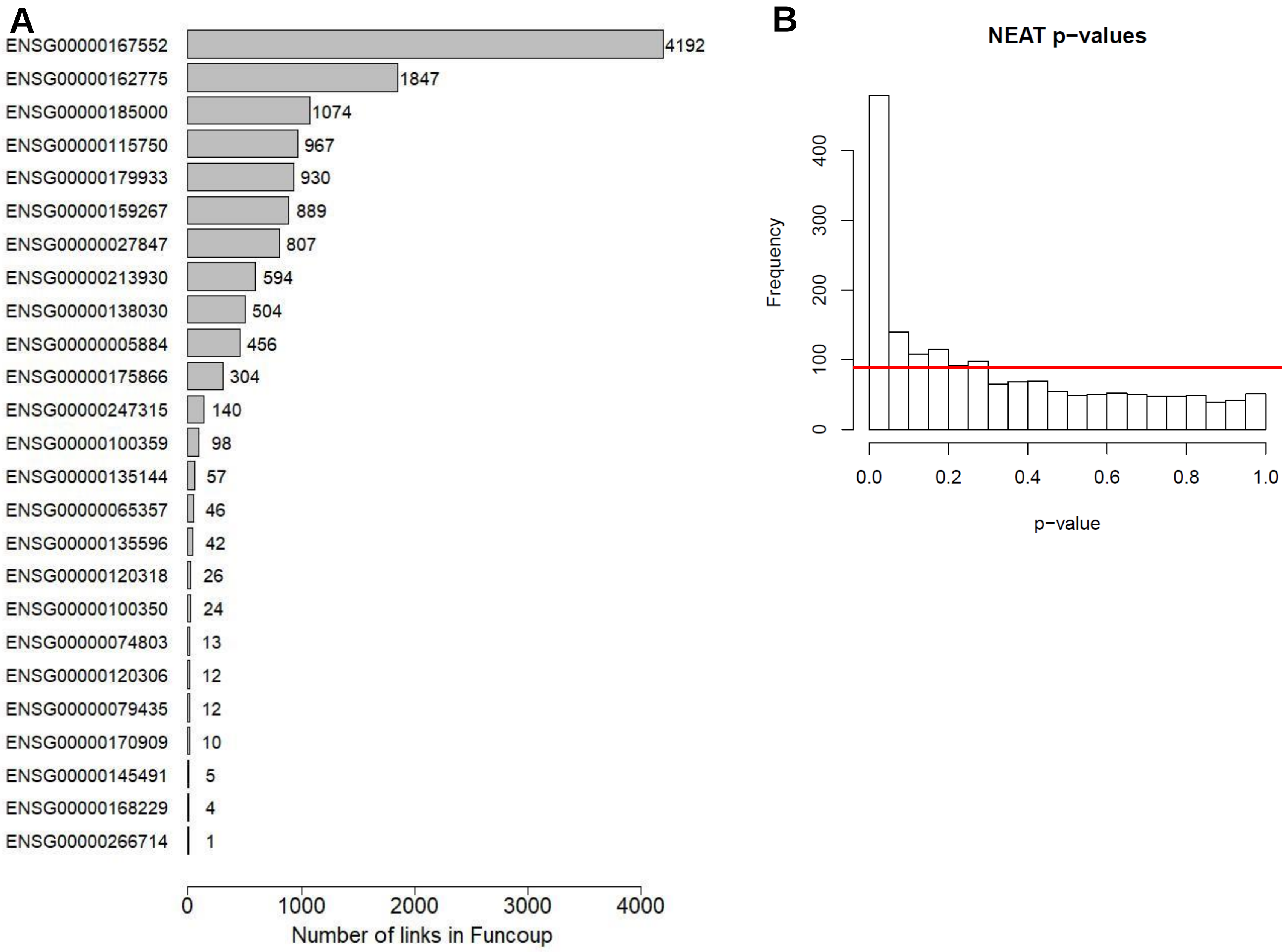

Supplementary Figure 2

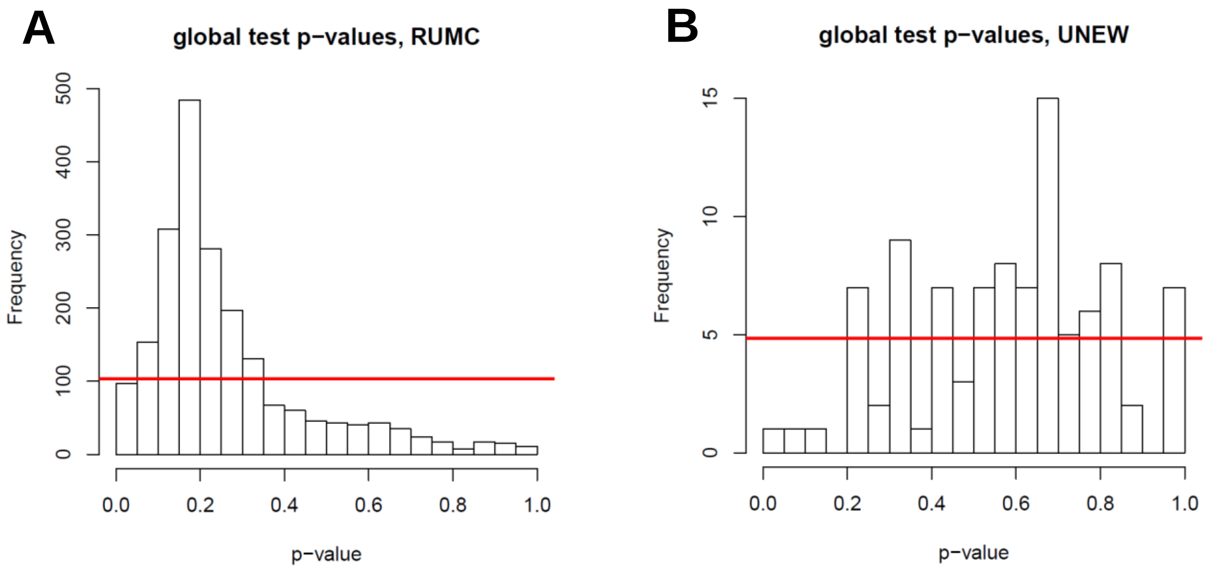

Supplementary Figure 3

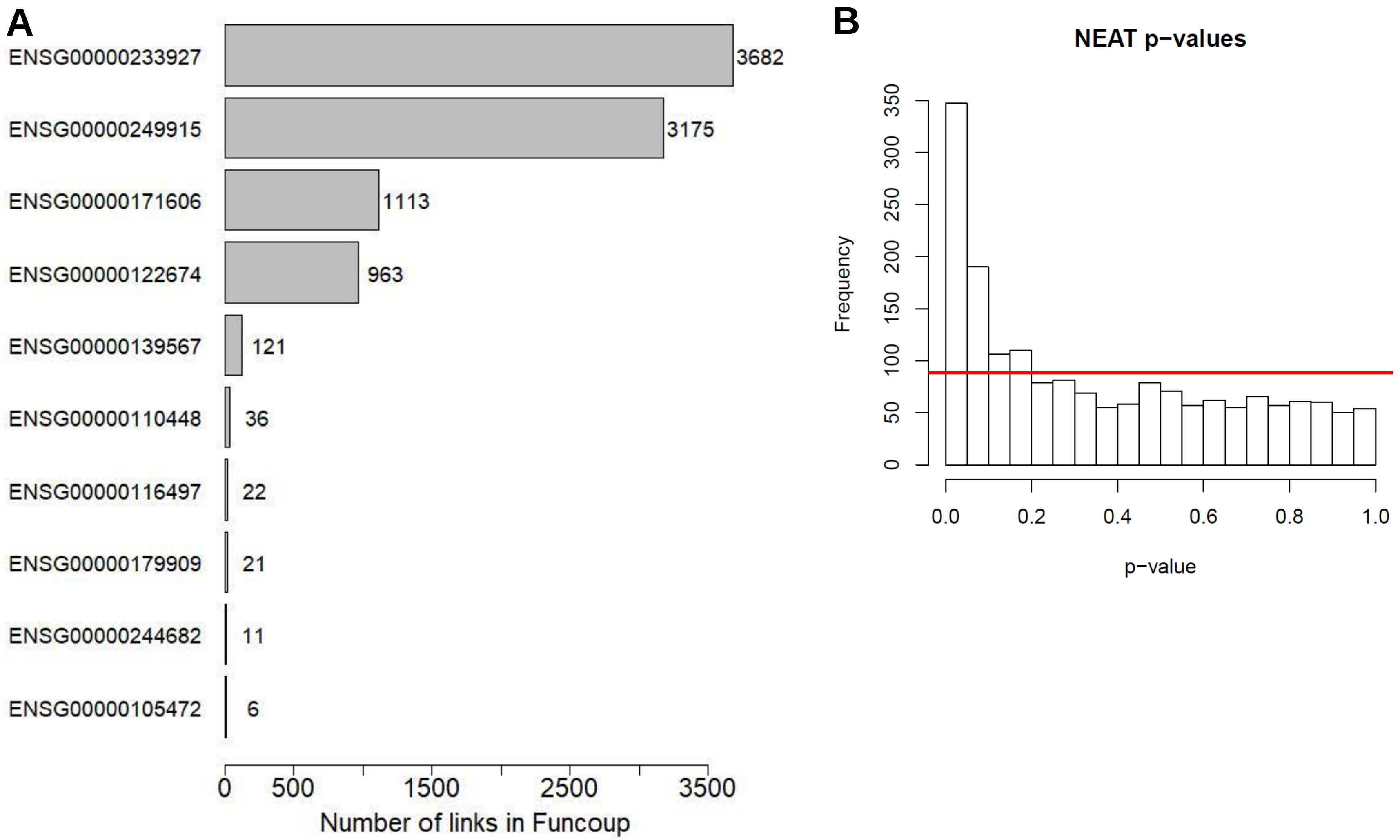

Supplementary Figure 4
