# Supplementary material for: Evaluation of blood gene expression levels in facioscapulohumeral muscular dystrophy patients
Source: Sci Rep. 2020 Oct 16;10:17547. doi: 10.1038/s41598-020-74687-5 (PMC7567883; doi:10.1038/s41598-020-74687-5)
Supplement: Supplementary file 3 — Supplementary Figure legends. [file 41598_2020_74687_MOESM3_ESM.docx]

**LEGENDS FOR SUPPLEMENTARY FIGURES**

Supplementary Figure 1. A-B) Histograms showing the distribution of the estimated percentage of each cell type in the RUMC (panel A) and UNEW (panel B) cohorts. C-D) Correlograms comparing pairs of estimated cell type percentages in the RUMC (panel A) and UNEW (panel B) cohorts.

Supplementary Figure 2. A) Distribution of the number of genes that each gene from the AGS1 gene set is connected to in the Funcoup network. B) Histograms of the distribution of the p-values from the NEAT test. The red horizontal line indicates the number of p-values that are expected to be < 0.05 in absence of network enrichment.

Supplementary Figure 3. Histograms of the distribution of the global test p-values in the RUMC (panel A) and UNEW (panel B) cohorts. The red horizontal line indicates the number of p-values that are expected to be < 0.05 in absence of any enrichment.

Supplementary Figure 4. A) Distribution of the number of genes that each gene from the AGS2 gene set is connected to in the Funcoup network. B) Histograms of the distribution of the p-values from the NEAT test. The red horizontal line indicates the number of p-values that are expected to be < 0.05 in absence of network enrichment.
